# Cannabidiol Reduces Aβ-Induced Neuroinflammation and Promotes Hippocampal Neurogenesis through PPARγ Involvement

**DOI:** 10.1371/journal.pone.0028668

**Published:** 2011-12-05

**Authors:** Giuseppe Esposito, Caterina Scuderi, Marta Valenza, Giuseppina Ines Togna, Valentina Latina, Daniele De Filippis, Mariateresa Cipriano, Maria Rosaria Carratù, Teresa Iuvone, Luca Steardo

**Affiliations:** 1 Department of Physiology and Pharmacology “Vittorio Erspamer”, Sapienza University of Rome, Rome, Italy; 2 Department of Pharmacology and Human Physiology, University of Bari, Bari, Italy; 3 Department of Experimental Pharmacology, University of Naples Federico II, Naples, Italy; Universidade Federal do Rio de Janeiro, Brazil

## Abstract

Peroxisome proliferator-activated receptor-γ (PPARγ) has been reported to be involved in the etiology of pathological features of Alzheimer's disease (AD). Cannabidiol (CBD), a Cannabis derivative devoid of psychomimetic effects, has attracted much attention because of its promising neuroprotective properties in rat AD models, even though the mechanism responsible for such actions remains unknown. This study was aimed at exploring whether CBD effects could be subordinate to its activity at PPARγ, which has been recently indicated as its putative binding site. CBD actions on β-amyloid-induced neurotoxicity in rat AD models, either in presence or absence of PPAR antagonists were investigated. Results showed that the blockade of PPARγ was able to significantly blunt CBD effects on reactive gliosis and subsequently on neuronal damage. Moreover, due to its interaction at PPARγ, CBD was observed to stimulate hippocampal neurogenesis. All these findings report the inescapable role of this receptor in mediating CBD actions, here reported.

## Introduction

Despite a significant increase in the understanding of the pathogenesis of Alzheimer's disease (AD) over the past two decades, therapeutic options for treating this condition are still very disappointing.

Depending on the heterogeneity of pathways that could initiate and drive sporadic AD, effective treatment for this illness rests on the ability to develop a multi-targeted approach, as used in current practice for other multi-factorial disorders [1]. According to this assumption, both natural and synthetic cannabinoids have been proposed as novel potential pharmacological tools able to blunt underlying disease processes, thus ameliorating symptoms and slowing down illness progression [2], [3].

Unfortunately, *Cannabis* derivatives are therapeutically limited by their unwanted psychotropic effects. However, one interesting exception to this is represented by cannabidiol (CBD), the major constituent of the plant, which lacks any undesired psychomimetic action. Converging evidence provided over the last years, also by our group, demonstrated that CBD may account for a significant reduction of β amyloid (Aβ) induced neuronal cell death, due to its ability to scavenge reactive oxygen species and reduce lipid peroxidation [4]. That CBD exerts anti-inflammatory properties, impairing the inducible form of nitric oxide synthase (iNOS) and interleukin 1β (IL-1β) expression which consequently decreases their release was also proved in an *in vivo* model of AD [5]. Moreover, CBD was reported to blunt τ hyperphosphorylation in cultured neurons by reducing phosphorylation of glycogen synthase kinase 3β (GSK3β), acting as a Wnt/β-catenin pathway rescuer, although alternative mechanisms may be implicated in inducing this effect [6]. Indeed, since GSK3β also promotes amyloid precursor protein (APP) processing, and so increasing Aβ generation [7], the CBD-mediated inhibition of GSK3β is likely to be effective in reducing the amyloid burden. Moreover, CBD was also described to protect neurons against glutamate toxicity [8], an effect occurring independently of the cannabinoid receptor 1 (CB1) signalling [9]. Despite such impressive properties and promising actions, the precise site at which CBD could exert its neuroinflammatory and neuroprotective effects is still not fully elucidated. The recently discovered ability of different cannabinoids, including CBD, to display an extra-cannabinoid receptor binding activity has been highlighted by the observation that these compounds may go nuclear to exert their activity through the interaction with peroxisome proliferator-activated receptors (PPARs) [10]. The PPARs belong to the family of nuclear hormone receptors and their activity is generally regulated by steroids and lipid metabolites. At present three different PPAR isoforms (PPARα, PPARβ, also called δ, and PPARγ) have been identified [11]; they have been reported to control the expression of genes related to lipid and glucose homeostasis and inflammatory responses [12]. A growing body of evidence suggests PPARs as drug targets for treating several dysmetabolic conditions and inflammatory degenerative diseases, as well. PPARγ is expressed in the CNS at low levels under physiological condition [13]. However, in some pathological situations, including AD, PPARγ expression, but not other isoforms, was shown to be elevated [14]. These findings suggested that PPARγ could play a role in regulating pathophysiological features of AD and established the basis for modulation of PPARγ activity in the treatment of the disease.

Therefore the present study was aimed at exploring whether CBD neuroprotective effects depend upon its activity on PPARs receptors, particularly on PPARγ isoform. To this purpose, the involvement of PPARs receptors in mediating anti-inflammatory and neuroprotective effects of CBD both *in vitro* in primary cultured astrocytes and *in vivo*, in a rat model of AD-related neuroinflammation induced by the intrahippocampal injection of fibrillar Aβ (1–42) peptide was evaluated.

## Results

### CBD blunted neuroinflammation sustained by astrocytes through PPARγ selective activation *in vitro* and *in vivo*

**Figure 1:**
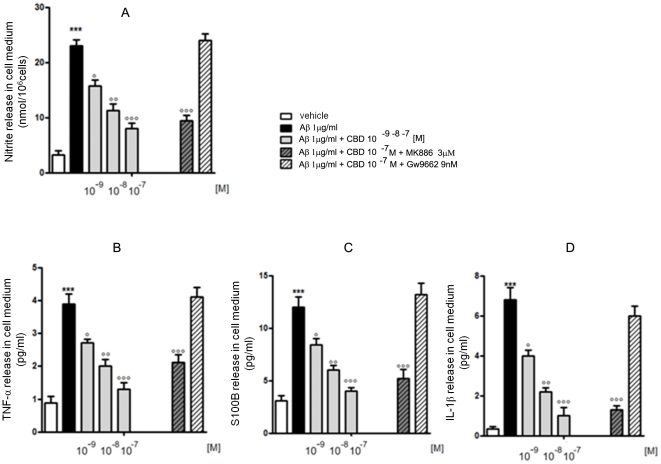
Effect of CBD on the release of inflammatory mediators by *in vitro* cultured astrocytes. Aβ-challenged astrocytes (1 µg/ml) were treated with CBD (10^−9^–10^−7^ M) in the presence of PPARα (MK886, 3 µM) or PPARγ (GW9662, 9 nM) antagonist. 24 h later, NO production was determined by measuring nitrite (NO_2_^−^) accumulation in the culture medium (A), whereas IL-1β, TNFα, and S100B release was determined by ELISA experiments (B,C,D). Each bar shows the mean ± S.E.M. of five separate experiments. ***p<0.001 vs. control; °p<0.05, °°p<0.01, and °°°p<0.001 vs. Aβ-challenged cells.

The first set of experiments was aimed at assessing the role of CBD (10^−9^–10^−7^ M) on the release of inflammatory mediators induced by Aβ challenge (1 µg/ml). Treatment with Aβ for 24 h resulted in a significant increase of NO, IL-1β, TNFα, and S100B release, as determined by Griess reaction and ELISA experiments (Figure 1). CBD concentration dependently antagonized the enhanced release of these pro-inflammatory molecules, and blockade of PPARγ with GW9662 (PPARγ antagonist, 9 nM) reversed this effect.

**Figure 2:**
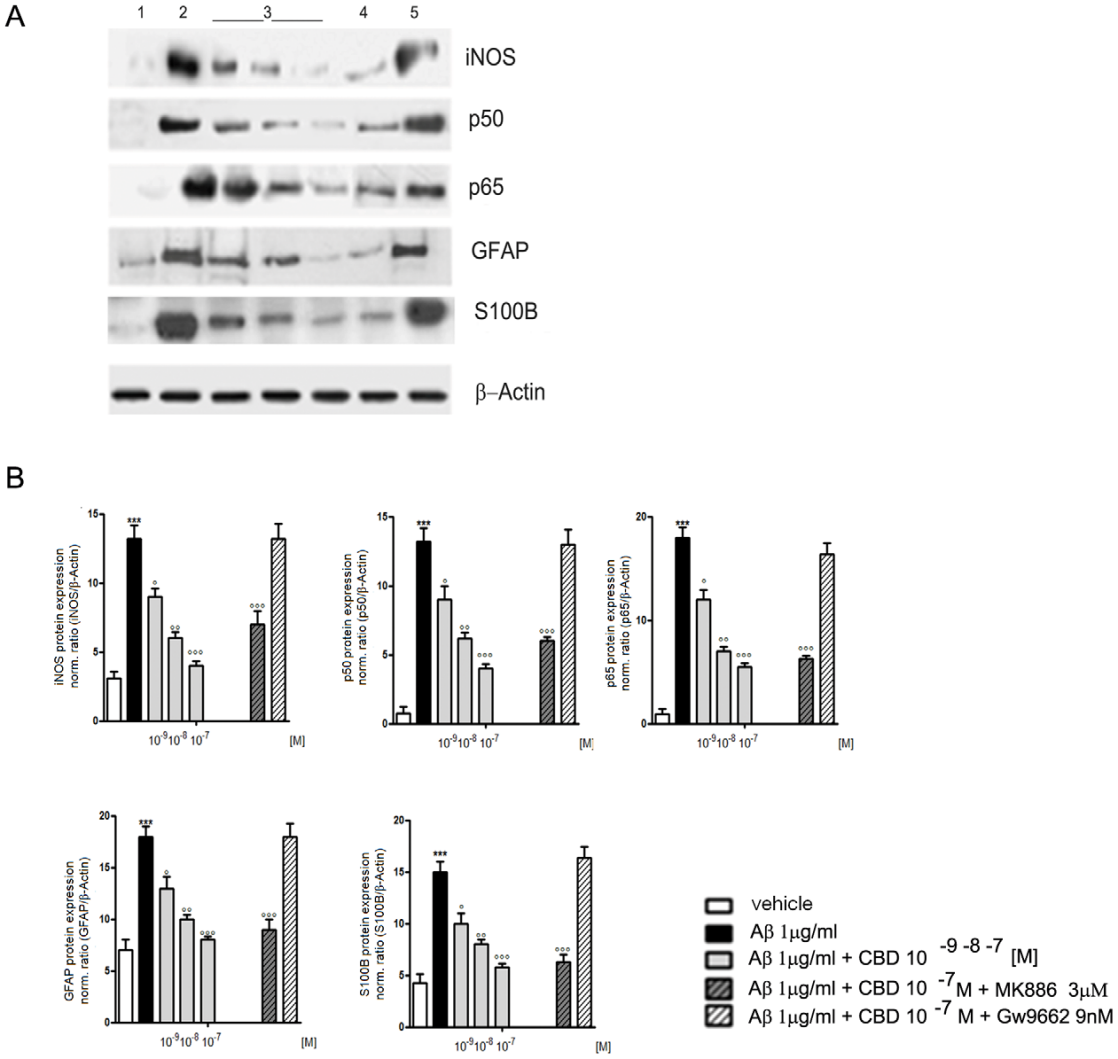
Effects of CBD on Aβ-induced iNOS, GFAP, S100B, and p50/p65 expression in rat astrocytes. Aβ-challenged cells (1 µg/ml) were treated with CBD (10^−9^–10^−7^ M) in the presence of PPARα (MK886, 3 µM) or PPARγ (GW9662, 9 nM) antagonist. iNOS, GFAP, and S100B expression was evaluated by Western blot 24 h after treatments. NF-κB activation was evaluated 3 h following treatments by Western blot analysis of its component p50 and p65. Figure shows results of Western blot analysis of proteins (A) and densitometric analysis of corresponding bands (B). Results are the mean ± S.E.M. of four separate experiments. ***p<0.001 vs. control; °p<0.5, °° p<0.01, and °°°p<0.001 vs. Aβ-challenged cells.

In astrocyte lysates, made 24 h after treatment, the parallel expression of iNOS, GFAP, and S100B was evaluated. CBD, in a concentration dependent manner, attenuated the Aβ-induced protein up-regulation and such an effect was related to the inhibition of NFκB. In fact, in astrocyte lysates prepared 3 h after treatment, CBD down-regulated the expression of both p50 and p65. The reduction of reactive gliosis displayed by CBD was to be considered as the result of a selective PPARγ dependent NFκB inhibition (Figure 2).

**Figure 3:**
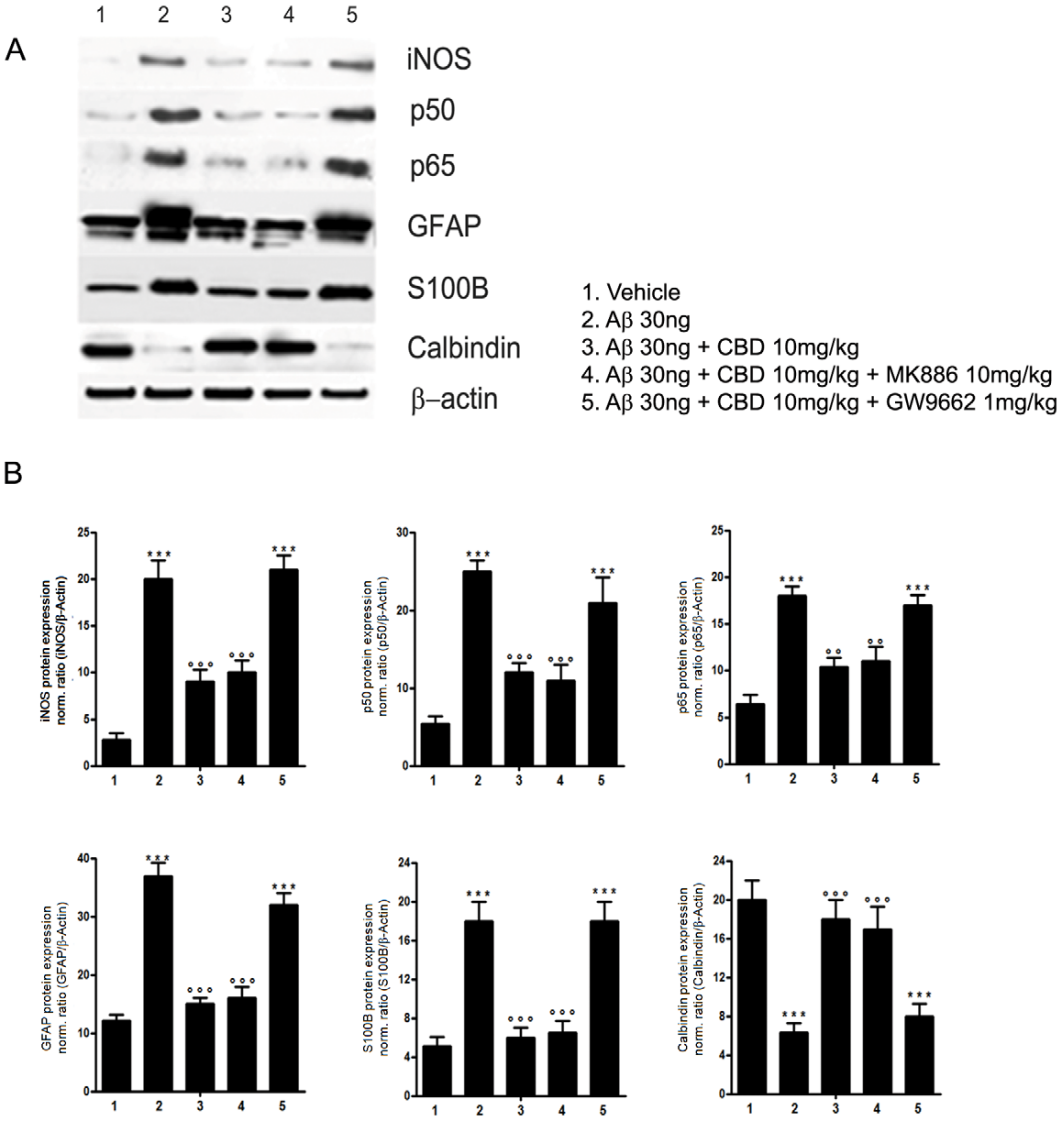
Effects of CBD on Aβ-induced iNOS, GFAP, S100B, calbindin, and p50/p65 expression in rat hippocampi. Results of Western blot analyses of iNOS, GFAP, S100B, calbindin, and p50/p65 performed on rat hippocampus homogenates (A) and densitometric analysis of corresponding bands (B). Results are the mean ± S.E.M. of three separate experiments. *** p<0.001 vs. vehicle inoculated rats; °°p<0.01 and °°° p<0.001 vs. Aβ-inoculated rats.

Results from hippocampal homogenates, isolated from rats Aβ-injected in the dorsal hippocampus and intraperitoneally administered with CBD in the presence or absence of MK886 (PPARα antagonist) and GW9662, demonstrated that Aβ induced the up-regulation of iNOS, GFAP, and S100B towards vehicle injected rats. CBD blunted Aβ effect through a reasonable inhibition of NFκB, as revealed by the parallel down-regulation of both p50 and p65. Moreover, CBD also counteracted the Aβ-induced calbindin down-regulation. All these observed effects displayed by CBD were caused by the selective activation of PPARγ (Figure 3).

### CBD administration inhibited reactive gliosis and rescued neuronal survival in Aβ-injected rat hippocampi through PPARγ activation

**Figure 4:**
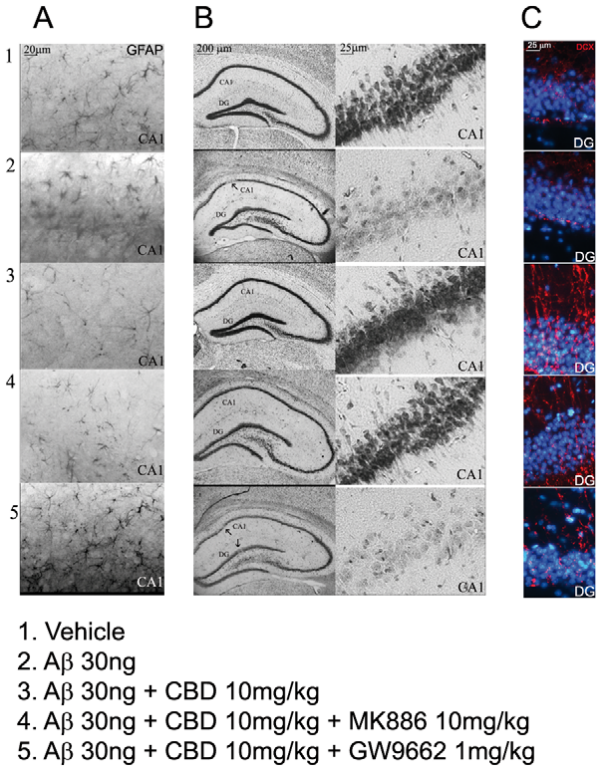
Effects of CBD on reactive gliosis, neuronal survival, and neurogenesis in rat hippocampi. A: representative photomicrographs of the CA1 area of rat hippocampus showing the results of immunohistochemical evaluation of GFAP. B: representative photomicrographs showing the results of Nissl staining of the whole rat hippocampus (2X magnification) and the corresponding CA1 region (10X magnification). C: immunofluorescence photomicrographs showing a particular (10X magnification) of DCX-labeled cells in the dentate gyrus (DG) of rat hippocampi.

*In vivo* experiments were aimed at assessing the potential modulating role of CBD on reactive gliosis and neuronal survival in rat hippocampi. Results from Nissl staining indicated that Aβ injection caused a severe neuronal loss, especially in CA1 area (site of injection), towards vehicle injected animals, while at the same time Aβ caused a marked astrocytic activation, as demonstrated by the increased expression of GFAP. According to *in vitro* results, *in vivo* 15 consecutive days administration of CBD (10 mg/kg) almost completely rescued CA1 pyramidal neurons integrity and this was accompanied by a massive down-regulation of gliosis entity as shown by a significant decrease of GFAP immunostaining. Once animals were treated with PPARγ antagonist (GW9662, 10 mg/kg) CBD neuroprotective functions resulted completely abolished (Figure 4 A, B).

Moreover, our results indicated that CBD treatment restored neurogenesis in dentate gyrus of Aβ-injected rat hippocampi through PPARγ selective activation.

The DG of rat hippocampi processed for DCX immunofluorescence revealed that Aβ treatment markedly reduced the entity of neurogenesis, as demonstrated by the reduction of DCX-immunopositive nuclei. CBD treatment was able to counteract Aβ-induced DCX depletion up to stimulate the basal neurogenesis versus controls. In line with the previously obtained results, CBD activity was unaffected by PPARα antagonism, whereas its effect was almost completely abolished by GW9662, the PPARγ antagonist (Figure 4, C).

## Discussion

Chronic neuroinflammation implicates protracted activation of both microglial and astroglial cells, with the consequent sustained release of pro-inflammatory molecules [15], [16], that act in an autocrine way to self-perpetuate reactive gliosis and in a paracrine way to kill neighboring neurons, thus expanding the neuropathological damage. Once started, neuroinflammation promotes neuronal death, powering a vicious cycle responsible for the progression of the pathology [17]. The possibility of interfering with this detrimental cycle by molecules which can reduce reactive gliosis has been proposed as a novel rationale to develop drugs able to blunt neuronal damage and consequently slow the course of AD.

Results from the present study prove the selective involvement of PPARγ in the anti-inflammatory and neuroprotective effects of CBD here observed either *in vitro* and *in vivo*. In addition, CBD significantly promoted neurogenesis in Aβ injured rat hippocampi, much expanding its already wide spectrum of beneficial actions exerted in AD models, a non negligible effect, due to its capability to activate PPARγ.

CBD was already reported to exert a marked anti-inflammatory effect through the A2A and 5HT1A receptors [18], [19], as well as to improve brain function [20]. In addition, it has been already demonstrated that CBD markedly downregulate reactive gliosis by reducing pro-inflammatory molecules and cytokine release that strongly occurs in Aβ neurotoxicity. This activity was linked to its ability to act as a potent inhibitor of NFκB activation induced by Aβ challenge [21]. The present findings, confirming the formerly obtained results and extending our knowledge about CBD pharmacology, indicate that a selective PPARγ activation occurs upstream to CBD-mediated NFκB inhibition. Such activation appears to be responsible for a large plethora of CBD effects. Indeed, the interaction of CBD at the PPARγ site results in a profound inhibition of reactive gliosis as showed by the reduction of both GFAP and S100B protein expression together with a marked decline of pro-inflammatory molecules and cytokine release observed in Aβ challenged astrocytes.

The *in vitro* findings were replicated in *in vivo* experiments, since once again CBD treatment resulted in a profound inhibition of astrocytic activation surrounding CA1 area Aβ-injected and induced a rescue of CA1 neuronal viability in comparison to control.

Along this line, results from hippocampal homogenates fully matched with *in vitro* data previously obtained from primary cultured astrocytes. Also in this case, CBD through the activation of PPARγ provoked a marked reduction of NO, TNFα, and IL-1β release in association with a parallel decline of GFAP, S100B, and iNOS protein expression. The observation that in the hippocampus the selective activation of PPARγ caused a decrease in p50 and p65 protein expression, further reinforces the importance of the sequence of events PPARγ activation/NFκB inhibition as responsible for the CBD anti-inflammatory effect. The PPARγ mediated inhibition of S100B induced by CBD represents a crucial step in interrupting self-perpetuation of the reactive gliosis cycle. Indeed, the over-release of this astroglial-derived neurotrophin actively exacerbates the pro-inflammatory cytokine loop fuelled by Aβ stimulation, massively accelerates amyloidogenicity by promoting cleavage of APP to Aβ and induces tau protein hyperphosphorylation, by disrupting the Wnt pathway [22]–[24].

Notably, both *in vivo* and *in vitro*, a selective involvement of PPARγ at the base of the neuroprotective and anti-gliotic effect of CBD appears peremptory, since a complete loss of any beneficial pharmacological activity of this phytocannabinoid occurs when it was co-administered with the PPARγ antagonist GW9662.

Neuroprotective effects exerted by PPARγ agonists in neuropathological conditions, including Aβ induced neuroinflammation and neurodegeneration have been largely described in the past years [25]. Besides microglial cells, emerging data indicate astrocytes and neurons as fundamental cell type targets for the beneficial role of PPARγ ligands [26]. Astrocytes represent the most abundant glial cells type in the CNS. Once these cells undergo reactive activation, they produce cytokines and other molecules involved in inflammatory response, which are thought to significantly contribute to expand brain damage. Interestingly astrocytes express the highest level of PPARγ in the CNS [27], [28], and accumulating evidence over the last decade indicate that PPARγ agonists may finely regulate their detrimental functions during a protracted activation exerting a profound anti-inflammatory and neuroprotective effects [29].

Such well-defined and highly effective beneficial activity due to PPARγ activation has recently demonstrated to functionally inactivate NFκB promoters inducing a massive down regulation of the activation of this transcription factor. Traditional PPARγ agonists like thiazolidinediones were reported to inhibit the overproduction of NO, IL-6, and TNF-α as well as the increased expression of the inducible enzymes iNOS and COX2 induced in LPS-stimulated astrocytic and microglial cultures [30]–[32]. Taken together all these findings fully match those concerning the anti-inflammatory and neuroprotective activities exhibited by CBD in this study.

AD neuroinflammation is a highly important feature that has been considered responsible for the progressive worsening of the disease. Given the tremendous complexity of AD, however, it appears to be oversimplified reasoning to consider only reactive astrogliosis and neuronal degeneration as the unique factors at the basis of the functional decline provoked by the course of the illness.

Neuropathological investigation has provided evidence that in AD brains progressive neuronal loss is not accompanied by a new neuronal replacement while alterations in neurogenesis have been reported to occur [33]. Both aspects are considered to be important in contributing to the long-term development of disease. Therefore the disease symptoms could partly be due to the impaired replacement of new hippocampal neurons from endogenous neuronal stem cells [34], [35], which is believed to promote learning and memory. Although there were some controversies over whether neurogenesis is increased or reduced in the pathogenesis of AD, later studies have confirmed deficient maturation of new neurons in AD patients [36]. As a result, an approach to enhance neurogenesis and/or maturation should be considered potential therapies for AD.

Moreover, activation of PPARγ has been reported to promote neurogenesis and agonists at these receptors regulate neuronal stem cell proliferation and differentiation as well [37]. In addition PPARγ activation promotes neurite outgrowth in mature neurons, significantly contributing to a proper neuronal connectivity in neuronal networks [38]. According to these observations, results from the current investigation demonstrate that CBD-mediated activation of PPARγ is associated with a significant neurogenic activity in the granule cell layer of the hippocampal DG. Results indicate that CBD markedly counteracts the massive reduction of neurogenesis in the DG caused by Aβ exposure towards control animals and this effect is consequential, as expected, to a selective PPARγ involvement.

It has been recently asserted that CBD could promote adult hippocampal neurogenesis by activating CB1 receptors [39]. Such an assumption was supported by the observation that the CBD neurogenic effect was lost in CB1 knock-out mice, suggesting that the pro-neurogenic action of this phytocannabinoid was clearly dependent on the interaction at the CB1 receptor, which shows a wide expression over the entire DG, including the neuronal precursor cells. However, the observation that Δ9-tetrahydrocannabinol, a preferential agonist at CB1 receptor site, failed in the same investigation to affect hippocampal neurogenesis, tempers the notion that CB1 receptor activation and CBD neurogenetic properties are linked in a straightforward way. Since CBD exhibits negligible affinity to CB1 receptors and the majority of actions appears to be cannabinoid receptor independent, it is possible to assume that the CBD effect on neurogenesis could only involve CB1 receptor sites indirectly.

In conclusion, results of the present research demonstrate that CBD may exert protective functions through a PPARγ dependent activation, which leads to a reduction in reactive gliosis and consequently in neurodegeneration. Moreover, in the current experimental conditions this phytocannabinoid appears to stimulate neurogenesis since it increases DCX immunopositive cell proliferation rate in rat DG.

Innovative therapeutic approaches which could significantly improve AD course require new molecules that will be able to have an impact on different pathological pathways, which converge at the progressive neurological decline. CBD has shown a capability to profoundly reduce reactive astrogliosis and to guarantee both direct and indirect neuronal protection in Aβ induced neuroinflammation/neurodegeration. So far, the lack of understanding of the precise molecular mechanism involved in CBD pharmacological actions, has had limited interest and has puzzled investigators. Currently, findings of the present study throw some light on the issue, and frame CBD as a new PPARγ activator.

## Materials and Methods

### Ethics Statement

All experiments were performed in accordance with the National Institutes of Health Guidelines for the Care and Use of Laboratory Animals (Institute of Laboratory Animal Resources, 1996) and those of the Italian Ministry of Health (D.L. 116/92), and they were approved by the Institutional Animal Care and Use Committees (Centro Servizi Veterinari-University of Naples Federico II; PROT. 012F).

### Astrocyte culture

Rat primary astroglial cultures were obtained from newborn Sprague-Dawley rats (two days old) according to Vairano et al. [40]. Brains were mechanically processed to obtain single cells that were seeded in 75 cm^2^ flasks at a density of 3×10^6^ cells/flask with 15 ml of culture medium (DMEM, 10% inactivated FBS, 100 IU/ml penicillin, 100 µg/ml streptomycin; all from Sigma Aldrich) and incubated at 37°C in a humidified atmosphere containing 5% CO_2_. Astrocytes were mechanically separated from microglia and oligodendrocytes, and plated onto 10 cm ø Petri dishes at a density of 1×10^6^ cells/dish or onto eight chambers polystyrene culture slides at a density of 3×10^4^ cells/chamber, depending upon the experimental procedure. Purity of cell cultures was tested by immunofluorescence with monoclonal anti-glial fibrillary acidic protein (GFAP) and only cultures with more than 95% GFAP-positive cells were employed for the experiments.

### Treatments in isolated primary astrocytes

Confluent primary astrocyte cultures were treated with 1 µg/ml Aβ (1–42) (Tocris Bioscience) in the presence or absence of the following substances: CBD (10^−9^–10^−7^ M), MK886 (3 µM), the selective PPARα antagonist, and GW9662 (9 nM), the selective PPARγ antagonist (all purchased from Tocris Bioscience). The concentrations of the substances were chosen according to the results of a series of pilot experiments aimed at identifying the lowest effective concentration (data not shown). No significant variation versus control was observed when CBD, MK886, or GW9662 were given alone (data not shown).

### Nitric oxide (NO) measurement

Production of nitric oxide (NO) was determined by measuring the amount of nitrite (NO_2_^−^) accumulated in supernatants of primary rat astroglial cells 24 h after treatments and in the inoculated ipsilateral hippocampi homogenates deriving from *in vivo* experiments. A spectrophotometer assay based on the Griess reaction was used [41]. The absorbance of controls and unknown samples was measured at 540 nm. The NO_2_^−^ concentration was thus determined using a standard curve of sodium nitrite (NaNO_2_) and referred to 1×10^6^ cells for the *in vitro* experiments or expressed as NO_2_^−^ referred to µg of homogenized hippocampal protein content for the *in vivo* experiments.

### ELISA

Quantitative determination of Tumor Necrosis Factor alpha (TNFα), interleukin 1β (IL1β), and S100B was carried out performing Enzyme Linked-Immuno-Sorbent Assay (ELISA) assays *in vitro* and in *ex vivo* deriving samples from homogenated hippocampi ipsilateral to the inoculation site; (TNFα and IL β kits purchased from Invitrogen; S100B kit from BioVendor). In *in vitro* experiments, 24 h after treatments, cell culture medium was collected and ELISA assay was performed according to kit instruction. Similarly, samples collected by hippocampal homogenates deriving from the *in vivo* experiments, also undergo ELISA.

### Western Blot analysis

Twenty-four hours after treatment, harvested astrocytes (1×10^6^ cells) were washed twice with ice-cold PBS, and collected by centrifugation at 180 *g* for 10 min at 4°C. Pellets were resuspended in 50 µl of ice-cold hypotonic lysis buffer (Tris/HCl pH 7.5 50 mM; NaCl 150 mM; EDTA 1 mM; Triton X-100 1%) supplemented with the proper protease inhibitor cocktail (Roche) and incubated on ice for additional 15 min. Ipsi- lateral hippocampi to injection site, deriving from *in vivo* experiments, were dissected from frozen excised rat brains and lysed with the same buffer. Cells or hippocampi were then mechanically lysed, and the total protein extracts were obtained by centrifugation at 13,000 *g* for 15 min at 4°C. Samples were subjected to SDS-polyacrylamide gel electrophoresis, and proteins were transferred onto nitrocellulose membrane and incubated with one of the following antibodies: anti-GFAP 1∶50000; anti-iNOS 1∶200; anti-p50 1∶400, anti p65 1∶1000, anti-calbindin 1∶1000, anti-S100B 1∶1000, anti β-actin 1∶1000 (all purchased from Abcam). After being extensively washed in TBS 1X with 0.1% Tween 20, the membrane was incubated for 2 h at room temperature with the proper secondary HRP-conjugated antibodies anti-mouse (1∶2000) or anti-rabbit (1∶3000; both purchased from Abcam). Finally, the membrane was developed by using enhanced chemiluminescence substrate (ECL from Invitrogen). Bands were revealed through a Versadoc (Bio-Rad Laboratories) and the corresponded digital images were analyzed with Quantity One Software (Bio-Rad Laboratories).

### Rats and Surgical Procedures

Adult male Sprague-Dawley rats (300–350 g) were obtained from Harlan Italy. They were housed in a pathogen-free barrier facility under a 12-h light/dark cycle, with ad libitum access to food and water. Rats (n = 40) were anesthetized i.p. with pentobarbital (60 mg/kg). They were then placed in a stereotaxic frame and inoculated with human Aβ (1–42) (Tocris Cookson) into the CA1 area (AP −3.3, ML −1.2, DV −2.6) The peptide was dissolved in artificial cerebrospinal fluid to the concentration of 10 µg/ml, and 3 µl was injected using a microdialysis pump, keeping the flow to the constant value of 0.5 µl/min. Control rats (n = 8) were treated according to the same procedure, and they were inoculated with an equivalent volume of artificial cerebrospinal fluid.

### *In Vivo* Treatments

After full recovery from stereotaxic surgery, depending upon the experimental protocol design, rats were i.p. administered for 15 days with: CBD 10 mg/kg, MK886 10 mg/kg, and GW9662 1 mg/kg. All coumpounds were dissolved in PEG/Tween80/saline (Sigma Aldrich), 5∶5∶90. Control rats were i.p. given an equivalent volume of the proper vehicle. All drug solutions were freshly prepared on the day of the experiment. The drug concentrations were chosen according to their IC50, and on the basis of the results of our preliminary experiments. Rats to be subjected to immunoblotting experiments underwent deep anaesthesia and sacrificed by decapitation. Their brains were extracted and ipsilateral hippocampi to injection site were dissected and frozen in liquid N_2_ before the tissue lysis for immunoblot analysis and/or ELISA assay. Rats to be subjected to morphological analysis were sacrificed through transcardiac perfusion with paraformaldehyde 4%. The extracted brains were post-fixed overnight with the same fixative, protected with 30% sucrose, and frozen using 2-methylbutane.

### Immunohistochemistry

Immunohistochemistry analyses were performed on hippocampal coronal sections adjacent to the site of the injection, obtained from both control and treated rats. Free-floating sections were treated with PBS containing 15 mM NaN_3_, 10% albumin, and 0.25% Triton X-100, and then they were incubated at 4°C in a damp chamber by continuous shaking with mouse anti-GFAP antibody (1∶400, Sigma Aldrich). After two overnight incubation, sections were treated with the biotinylated secondary antibody (1∶200; Vector Laboratories) and with the preformed avidin-biotinylated peroxidase complex (VECTASTAIN ABC kit; Vector Laboratories), and the reaction was revealed by 3,3-diaminobenzidine tetrahydrochloride (Sigma Aldrich). Sections were mounted upon gelatin-coated slides before coverslipping with a non-aqueous medium.

### Immmunofluorescence

In the same experimental conditions above described, immunofluorescence analyses were performed on hippocampal coronal sections adjacent to dentate gyrus (DG). Briefly, slices derived from both control and treated rats were blocked in 10% albumin bovine serum 0.1% Triton-PBS solution for 90 min and subsequently incubated for 1 h with a 10% albumin bovine serum 0.1% Triton-PBS solution of anti-doublecortin (DCX) antibody (dil. 1∶250 v/v). DCX antibody was purchased from Cell Signaling. Sections were incubated for 1 h in the dark with the proper secondary antibody: Tetramethyl Rhodamine Isothiocyanate (TRITC) conjugated anti-rabbit 1∶100 (both from Abcam). Pictures were taken using a camera (Nikon DIGITAL SIGHT DS-U1) connected with a microscope (Nikon ECLIPSE 80i by Nikon Instruments Europe) provided of the proper fluorescent filters. Slides were thus analyzed with a microscope (Nikon ECLIPSE 80i by Nikon Instruments Europe), and images were captured at 10X magnification by a high-resolution digital camera (Nikon DIGITAL SIGHT DS-U1).

### Statistical Analysis

Results were expressed as mean ± S.E.M. of experiments. Statistical analysis was performed using parametric one-way analysis of variance, and multiple comparisons were performed by Bonferroni's test. Values of *p<0.05* were considered significant.
